# Incidence of Prediabetes and Type 2 Diabetes among People Aged over 20 Years in Ahvaz: A 5-Year Perspective Study (2009–2014)

**DOI:** 10.1155/2016/4908647

**Published:** 2016-11-28

**Authors:** Seyed Mahmoud Latifi, Majid Karandish, Hajieh Shahbazian, Leila Hardani Pasand

**Affiliations:** ^1^Health Research Institute, Diabetes Research Center, Ahvaz Jundishapur University of Medical Sciences, Ahvaz, Iran; ^2^Nutrition and Metabolic Diseases Research Centre, Ahvaz Jundishapur University of Medical Sciences, Ahvaz, Iran

## Abstract

*Background*. The present study is the fourth cohort study conducted in the Middle East on the evaluation of prediabetes and type 2 diabetes, implemented in Ahvaz, Iran.* Methodology*. The individuals aged over twenty years who had participated in a study on the prevalence of metabolic syndrome in 2009 (Phase 1) in Ahvaz were invited again in 2014. The questionnaires were completed via interview, and anthropometric parameters were measured by standard method. The logistic regression and chi-square test were used for data analysis.* Results*. In the median of five-year follow-up, a number of 593 people participated in reexamination from which 396 individuals were nondiabetic in Phase 1. The incidence of diabetes and prediabetes was 21.9 and 40.6 per 1000 person-years, respectively. Among Phase 1 prediabetics, 16.8% were diagnosed with diabetes in a five-year period. The factors affecting the incidence of prediabetes among the people younger than 65 years include age, family history of diabetes, and gender. The age factor plays an important role in the transformation of prediabetes to diabetes.* Conclusion*. The city of Ahvaz with type 2 diabetes incidence of 13.64 per 1000 person-years is one of the areas with high incidence of diabetes in Iran.

## 1. Introduction

Type 2 diabetes defined as non-insulin-dependent or adult-onset diabetes originates from the body's inability to consume insulin which is frequently accompanied with weight gain and physical inactivity. As one of the most costly and prevalent chronic diseases worldwide, diabetes is constantly increasing due to changes in lifestyle and improvement of public health status which leads to higher rate of survival [1]. According to the World Health Organization report in 2012, it is estimated that diabetes account for 3.5% mortality from noncommunicable diseases. Moreover, it results in 22% mortality from cardiovascular diseases and 16% mortality from heart attack cases [2]. The majority of recent instances of diabetes have been reported in developing countries. It has been predicted that the prevalence of diabetes will increase to the greatest level in the Middle East among all regions by 2030 [3]. The incidence of diabetes has been estimated to be 29% in United Arab Emirates [4], 16% in Oman [5], and 14% in Phase 1 of Tehran Lipid and Glucose Study [6]. The International Diabetes Federation (IDF) in 2014 reported 8.6% incidence of diabetes in adults (between 20 and 79 years of age) which is equal to 4.5 million people in Iran [7]. The prevalence of diabetes with the adjusted world population was estimated to be 8% in 2010 [8]. The total cost of diabetes in that year was also estimated to be US$ 600 million [9]. The perspective studies have the preference of revealing prevalence and incidence of disease, risk factors, and primary symptoms at the same time. The first report of diabetes incidence rate based on population, the Tehran Lipid and Glucose Study, in nondiabetic population aged over 20 years with the average of 3-year follow-up shows 11.8 per 1000 person-years. Therefore, approximately, 1.2% of Tehrani persons aged over 20 years are diagnosed with diabetes each year [10]. This 9-year report showed that the incidence of diabetes was up to 1% and that there is an intense increase of prediabetes condition which is up to 4% in community population per year [11]. The incidence of diabetes and prediabetes in a 7-year cohort study in Isfahan was 18.9 and 32.3 per 1000 person-years, respectively [12].

The incidence and prevalence of diabetes in rural population aged over 30 years in Iran (Kalale town) in 2005 were reported to be 0.6 and 1.13%, respectively [13]. The crude incidence of type 2 diabetes in England, at the time interval of 1991–2010, was equal to 515 per 100 000 population [14]. In American population aged over 20 years, 1.7 million individuals with new cases of type 2 diabetes and 86 million with prediabetes were diagnosed in 2012 which is higher than the statistics of 79 million people in 2010 [15]. In the follow-up of an 11-year study including Aboriginal Australian residents implemented on 686 individuals aged between 20 and 76 years, the incidence of diabetes was increased from 2.2 in population younger than 25 years to 39.9 per 1000 person-years in the age range of 45–54 years [16]. The first perspective study in Ahvaz, capital of Khuzestan province located in south west of Iran, was implemented so as to assess the incidence of metabolic syndrome and diabetes in two phases of 2009 and 2014 [17]. It is worth mentioning that Ahvaz being endowed with warm climate condition and diverse ethnicities of Arab, Fars, Lor, and others is considered as a good sample of the demographic profile of the Khuzestan province.

## 2. Research Design and Methods

The first phase of prevalence of metabolic syndrome and its related factors was implemented in 2009 in Ahvaz by the Diabetes Research Center, Ahvaz Jundishapur University of Medical Sciences [12]. The target population includes residents aged over 20 years in Ahvaz. The sampling was carried out via cluster sampling method by inviting the participants to selected health centers in Ahvaz. A questionnaire that includes age, sex, marital status, ethnicity, education level, family history of diabetes mellitus (DM), hypertension (HTN) and obesity, smoking and parity, and previous history of gestational diabetes mellitus in women were filled for all of the participants. Moreover, blood pressure, weight, height, body mass index (BMI) [weight (kg)/height (m)^2^], and abdominal and waist circumference were measured in each participant. Blood pressure was measured by a standard sphygmomanometer after 15 min rest in a sitting position. The cuff was placed on the right arm at the heart level and then the device was quickly pushed until 30 mmHg above radial pulse disappearance. Blood pressure was measured twice with at least 30 min interval between two measurements and mean of these two measurements was taken as blood pressure. Anthropometric measurements were performed after removing shoes and wearing a light dress. Weight and height were measured according to the standard program. Waist circumference was measured at the midpoint between the lowest rib and the upper lateral border of the right iliac crest and hip circumference at the point of maximum hip diameter. After 12 h of fasting, blood samples were taken in the morning. Sample was centrifuged, and serum was isolated and stored in the refrigerator. Thereafter, it was sent to Diabetes Research Center laboratory. Triglyceride (TG), fasting blood sugar (FBS), cholesterol, and high density lipoprotein (HDL) were measured using an enzymatic colorimetric method with Pars Azmoon kit (Iran) (with Biotechnical Instruments model BT-3000, Germany).

The second phase was repeated five years later in 2014. The participants were recalled by health centers executing the first phase, and the adjustment questionnaires, experiments, and anthropometric measurements were performed as in Phase 1. A total number of 593 individuals possess complete information.

The variables observed in this regard are as follows:

Diabetes and prediabetes: based on definition presented by American Diabetes Association in 1997, FPG ≥ 126 mg/d is considered diabetic (or the same rate with medication) and 100 ≤ FPG < 126 mg/d is regarded as prediabetes [18].
BMI: BMI < 25 kg/m^2^ is normal weight, 25 ≤ BMI ≤ 29.99 is overweight, and BMI ≤ 30 kg/m^2^ is obesity.
Blood pressure disease: blood pressure (bp) is ≥140/90 mmHg (or the same rate with medication).
Abdominal obesity: waist circumference (WC) is <102 or WC > 88 cm in men and women, respectively [18].
Family history of diabetes: It is affliction with type 2 diabetes in at least one of the parents or brothers or sisters of the person.

## 3. Results

Among all of the 593 participants who took part in the second phase of the study, 282 individuals are men (47.6%) and 311 individuals are women (52.4%). The mean age in the beginning stages was 42.7 ± 13.4 years. The prevalence of diabetes and prediabetes was equal to 15.2 and 22.6% in 2009, respectively, which afterwards reached 20.9 and 18.3% in the second phase. The incidence of diabetes among healthy individuals present in Phase 1 is 21.9 per 1000 person-years and prediabetes incidence is 40.8 per 1000 person-years. The mean age of healthy individuals in Phase 1, who later became affected by diabetes, is 51.7 ± 11.24, while that of prediabetes patients is 46.55 ± 12.5 and that of healthy people is 38.34 ± 12.7 years. The incidence of type 2 diabetes among prediabetes individuals present in Phase 1 is 34.5 per 1000 person-years. In a 5-year period, of all prediabetics in phase 1, 50.2% became healthy, 33.1% remained prediabetic, and 16.8% changed to diabetics.

The mean age of healthy individuals in Phase 1, who later became diabetics in the course of 5 years, is 51.7 ± 11.24. The mean age of the ones who became prediabetics is 46.55 ± 12.5 and that of the ones who remained healthy is 38.34 ± 12.7 years.

According to Table 1, these variables are significantly different: age, BMI, family history of diabetes, and abdominal obesity among genders (*p* ≤ 0.013).

As seen in Table 2, the incidence of diabetes in Ahvaz shows an ascending trend with age, and it starts increasing from 4 in 20–29 years to 84.2 in 60–69 years (decreasing above 70 years). The incidence of prediabetes increases from 25.2 in the range of 20–29 years to 54.5 in 50–59.

The highest level of diabetes and prediabetes incidence was among the participants of 60–69 and ≥70 years, respectively. The incidence of diabetes in Ahvaz population starts from 10.03 in people with normal weight to 21.05 in overweight people and 46.03 of obese people in whom the risk of diabetes infliction is higher than five times.

As seen in Table 3, the number of people with diabetes has increased from 63 (10.8%) in Phase 1 to 88 (15.1%) in Phase 2, whereas the number of healthy people has decreased from 394 (67.8%) in Phase 1 to 370 (63.7%) in Phase 2. The number of people with prediabetes has only shown a minor difference.

The variables including age, blood pressure, abdominal obesity, family history of diabetes, and BMI are closely related to the incidence of diabetes, and ethnicity is related to the incidence of prediabetes. The mentioned variables were inserted in a multiple logistic regression model.  Table 4 shows the factors affecting the incidence of diabetes and prediabetes among healthy people in Phase 1.

## 4. Discussion

In the cohort study implemented in two phases among people aged over 20 years in 2009 and 2014 in Ahvaz, incidence of diabetes was calculated as 22.03 per 1000 person-years in healthy people of Phase 1. The incidence of diabetes among men from Ahvaz was 22.03 and in women was 21.78 (per 1000 person-years). In a cohort study with median of 6- and 9-year follow-up in population aged over 20 years in Tehran Lipid and Glucose Study (TLGS) during two time periods (2001–2005 and 2001–2009), the incidence of diabetes was 11.9 and 10.6 per 1000 person-years in nondiabetic individuals, respectively [19]. The incidence of diabetes in Ahvaz (22.03) was higher than that of the two studies in Tehran (11.9 and 10.6 per 1000 person-years). There was no significant difference in the incidence of diabetes between men and women from Ahvaz (*p* = 0.96) which is the same with the Tehran study (10.2 for men and 11 for women per 1000 per person-years) [20]. The reason for higher level of diabetes incidence in Ahvaz compared to Tehran can be attributed to the extremely hot climate in a period of more than half of the year which limits the residents' activity, type of nutrition, and lifestyle. Besides, the time difference in both studies is another reason for higher diabetes incidence in Ahvaz compared to Tehran.

In the 10-year cohort study in India, the overal incidence of diabetes was 22.2 (per 1000 person-years), 22.3 in men and 24.5 in women [21]. In another cohort study from 1997 to 2005 in India, the incidence of diabetes was 20.2 (per 1000 person-years) [22]. Moreover, in a study including the Aboriginal Australian residents aged from 20 to 70 years, the incidence of diabetes was 18 (per 1000 person-years) [16]. These three reported diabetes incidences are close to that of Ahvaz. In the following cohort studies the diabetes incidence was lower than the present one. The diabetes incidence in America in 2012 was 7.8 (per 1000 person-years) [15], in England in 1991–2010 was 5.15 (per 1000 person-years), and was 8 in Taiwan (per 1000 person-years) in 2007 [23].

The incidence of diabetes in Ahvaz shows ascending trend with age; this result is the same as that of the study in Tehran. In Aboriginal Australians study, the incidence rates increased with increasing age, from 2.2 per 1000 person-years for those younger than 25 years to 39.9 per 1000 person-years for those aged 45–55 years which is the same as Ahvaz study [15]. The incidence of diabetes of Ahvaz population starts from 10.03 (per 1000 person-years) in people with normal weight to 21.05 in overweight people and 46.06 in obese people where the risk of diabetes infliction is approximately five times. Moreover, it increases from 41 to 86 and 110 (per 1000 person-years) in the study in Tehran which has an increasing trend similar to the present study; however, in this study, the range between the three weights is less than that of the study in Tehran. On the other hand, in Aboriginal Australians study, incidence of diabetes in three weights was within the range of 8.6, 21.2, and 53.2%, respectively, close to the results in this study [15].

The incidence of diabetes in Ahvaz population with family history of diabetes was 37.3 (per 1000 person-years) which is twice more than the individuals with no record of diabetes (16.5 per 1000 person-years). In comparison the incidence of diabetes in the study in Tehran was 129 compared to 99 (per 1000 person-years) [13]. The family history of diabetes shows that the risk of diabetes in Ahvaz was higher than 1.7 times and in Tehran it was 2.4 times more than people with no family history of diabetes which means that the risk of diabetes in Tehran is higher than that in Ahvaz.

Ahvaz study shows the risk of diabetes to be 6.37 in people with abdominal obesity and Tehran study shows that it is 1.4 times more compared to the people with no abdominal obesity. Besides, high blood pressure in Ahvaz causes the risk of diabetes to be 2.5 while it was 1.9 times higher in Tehran compared to people with no high blood pressure. The risk of abdominal obesity and high blood pressure in Ahvaz were higher than those in Tehran.

The incidence of prediabetes in this study is 40.7 per 1000 person-years, 41.7 in males and 39.9 in females per 1000 person-years. The incidence of prediabetes in Isfahan study was 32.3 per 1000 person-years, which is less than this survey [12]. Each year, 4% of the total adult population in Tehran change from healthy glucose to prediabetes condition [11]. The incidence of prediabetes in Tehran study was 46.1 and 36.8 per 1000 person-years in men and women, respectively [24]. The incidence of prediabetes among males and females in Ahvaz does not show a significant difference, whereas this difference in Tehran study is significant, with incidence of prediabetes in men being higher than women. The study implemented in India showed that the incidence of prediabetes in men is about 31 and it is 34 in women (per 1000 person-years) which is lower compared to Ahvaz with regard to both genders.

The incidence of prediabetes among healthy people in Phase 1 changes from 25.2 in ages 20–29 years to 54.5 in ages 50–59 years and also 114.3 in ages higher than 70 years as the age increases which signifies the ascending trend with age. Moreover, the incidence of prediabetes among non-Arab people with *p* = 0.03 is significantly more compared to Arab ethnicity. The study shows that the incidence of diabetes among prediabetic people in Phase 1 is estimated to be 40.8 (per 1000 person-years) which has great differences with the study in India (78.9 per 1000 person-years). The incidence of diabetes in the participants of Phase 1 affected by prediabetes among men is more than that in women which changes from 22.2 per 1000 person-years within the age range of 20–29 to 120 with age 60–69 years.

The multiple logistic regression shows that age, BMI ≥ 30, and family history of diabetes affect the incidence of diabetes, which is inconsistent with the 6-year cohort study in Tehran. However, the incidence of prediabetes is only related to age.

## 5. Conclusion

The present study shows that among people aged over 20 years in southwest of Iran, the incidence of diabetes was 21.9 per 1000 person-years and the incidence of prediabetes was 40.8 per 1000 person-years which is higher than in other studies formerly implemented in Iran. The men population is exposed to higher level of risk when compared to women.

## Figures and Tables

**Table 1:** Demographic characteristics of study population by gender.

	Total 593 (100%)	Female 311 (100%)	Male 282 (100%)	*p* value

Age				
20–29	25.5	51.5	48.5	0.039
30–39	25.8	64.7	35.3	
40–49	22.7	57.8	42.2	
50–59	17.9	7.9	52.1	
60–69	5.8	59.1	40.9	
≥70	2.3	22.2	77.8	
BMI				
Normal	32.3	27.3	38.3	0.0001
Overweight	39.8	35.7	42.2	
Obese	28.8	37	19.5	
Family history of diabetes				
Positive	28.6	33	23.7	0.013
Negative	71.4	67	76.3	
Ethnic				
Arab	53.3	53.4	53.2	0.96
Fars	46.7	56.6	46.8	
Abdominal obesity				
Normal	72.5	87.9	58.5	0.0001
Abnormal	27.5	12.1	41.5	
Blood pressure				
Normal	85.3	84.2	86.5	0.43
Abnormal	14.7	15.8	13.5	

**Table 2:** The incidence of diabetes and prediabetes among healthy people of Phase 1 and the Incidence of diabetes in people with prediabetes by demographic variables.

	Healthy people in Phase 1			Prediabetes people in Phase 1
	Incidence of diabetes	Odds ratio (95% CI)	Incidence of prediabetes	Incidence of diabetes
Sex				
Male	22.03	1	41.7	66.7
Female	21.78	1.4 (0.6–3.14)	40	38.2
*p *value	0.96	—	0.94	0.18
BMI				
<25	10.03	1	46.9	63.1
25–29.99	21.05	2.08 (0.62–6.9)	33.9	34.5
≥30	46.06	5.75 (1.8–18.3)	43.4	61.2
*p *value	0.0004	—	0.45	0.37
Age				
20–29	4	1	25.2	22.2
30–39	10.06	2.55 (0.48–13.5)	38.7	47.05
40–49	31.1	8.35 (1.83–38.1)	47.3	93.86
50–59	27.45	11.1 (2.4–50.9)	54.5	105.26
60–69	84.2	26.4 (5.1–136.35)	27.9	120
≥70	23.8	6.18 (0.5–0.75)	114.3	44.4
*p *value	**0.0002**	—	0.06	0.12
Family history of diabetes				
No	16.5	1	39.8	42.1
Yes	37.3	1.73 (0.74–4.03)	41.1	79.5
*p *value	0.005	—	0.99	0.11
Ethnicity				
Arab	19.3	1	31.9	51.8
Fars	25.8	1.06 (0.47–2.37)	53.6	52.2
*p *value	0.21	—	0.03	0.86
Abdominal obesity				
No	15.5	1	38.3	47.5
Yes	49	6.37 (2.93–15.47)	49	60
*p *value	0.0001	—	0.42	0.66
Hypertension				
No	17.2	1	36.7	37.8
Yes	40.2	4.5 (1.87–10.76)	53.6	80
*p *value	0.0001	—	0.16	0.055

**Table 3:** Frequency cross-table of diabetics and prediabetics in baseline and after 5 years.

Phase 1	Phase 2			
	Diabetes	Prediabetes	Healthy	Total
Diabetes	42 (7.2)	9 (1.5)	12 (2.1)	63 (10.8)
Prediabetes	21 (3.6)	41 (7.1)	62 (10.7)	124 (21.3)
Healthy	25 (4.3)	73 (12.6)	296 (50.9)	394 (67.8)
Total	88 (15.1)	123 (21.2)	370 (63.7)	581 (100)

**Table 4:** Factors affecting the incidence of diabetes, prediabetes in healthy people of Phase 1 by logistic regression.

Healthy to diabetes variables	Odds 95% CI	*p *value

Age (year)	1.08 (1.04–1.11)	0.0001
BMI (≥30/<25) (kg/m^2^)	3.64 (1.16–11.4)	0.026
Family history of diabetes (yes/no)	3.64 (2.05–5.29)	0.0001
Healthy to prediabetes age	1.025 (1.004–1.047)	0.021
Prediabetes To diabetes age	1.035 (1.001–1.07)	0.023
